# The Impact of Genetic Polymorphism on Complication Development in Heart Failure Patients

**DOI:** 10.3390/jcm14010035

**Published:** 2024-12-25

**Authors:** Madina R. Zhalbinova, Saule E. Rakhimova, Ulan A. Kozhamkulov, Kenes R. Akilzhanov, Nurlan K. Shaimardanov, Gulbanu A. Akilzhanova, Joseph H. Lee, Yuriy V. Pya, Makhabbat S. Bekbossynova, Ainur R. Akilzhanova

**Affiliations:** 1 National Laboratory Astana, Nazarbayev University, Astana 010000, Kazakhstan; madina.zhalbinova@nu.edu.kz (M.R.Z.); saule.rakhimova@nu.edu.kz (S.E.R.); ulan.kozhamkulov@nu.edu.kz (U.A.K.); 2 Eurasian Society of Personalize Medicine, Astana 010000, Kazakhstan; 3 Department of Medicine, Semey Medical University, Pavlodar Branch, Pavlodar 140000, Kazakhstan; a_kenes79@mail.ru (K.R.A.); gulbanu-25@mail.ru (G.A.A.); 4 Department of Medicine, Semey Medical University, Semey 071400, Kazakhstan; abylaikhan@gmail.com; 5 Sergievsky Center, Taub Institute, Columbia University Medical Center, New York, NY 10032, USA; jhl2@cumc.columbia.edu; 6 National Research Cardiac Surgery Center, Astana 010000, Kazakhstan; pya_yuriy@mail.ru (Y.V.P.); mcardio_s@mail.ru (M.S.B.); 7 Department of General Biology and Genomics, L. N. Gumilyov Eurasian National University, Astana 010000, Kazakhstan

**Keywords:** genotype, polymorphism, personalized treatment, heart failure, left ventricular assist device (LVAD)

## Abstract

**Background:** Despite the high progress that has been made in the field of cardiology, the left ventricular assist device (LVAD) can still cause complications (thrombosis/bleeding) in heart failure (HF) patients after implantation. Complications develop due to the incorrect dose of antithrombotic therapy, due to the influence of the non-physiological shear stress of the device, and also due to inherited genetic polymorphisms. Therefore, the aim of our study is to identify the influence of the genetic polymorphisms on complication development in HF patients with implanted LVADs with prescribed antiplatelet therapy. **Methods:** Our study investigated 98 HF patients with/without complications who were genotyped for 21 single-nucleotide polymorphisms (SNPs) associated with cardiovascular events, the coagulation system, and the metabolism of warfarin and aspirin drugs. This study performed a more detailed analysis on genetic polymorphism in the UGT1A6 gene and its influence on aspirin dose. **Results:** SNP rs2070959 in the *UGT1A6* gene showed a significant association with the group of HF patients with complications [(OR (95% CI): 4.40 (1.06–18.20), *p* = 0.044]. The genetic polymorphism of rs2070959 in the UGT1A6 gene showed a significant association in HF patients who received aspirin treatment on the 12th month after LVAD implantation [OR (95% CI): 5.10 (1.31–19.87), *p* = 0.018]. Moreover, our genotype distribution analysis showed that the GG genotype of rs2070959 in the UGT1A6 gene was significantly higher in the group with aspirin treatment than without treatment after the 12th month of treatment (50.0% vs. 0%, *p* = 0.008), especially in the group of patients with complications. A higher frequency of the GG genotype with long-lasting aspirin therapy up to the 12th month showed that 100 mg of aspirin was not an effective dose in the group of patients with complications. **Conclusions:** Our study identified that genotyping for genetic polymorphism rs2070959 in the UGT1A6 gene could predict the recommended dose of aspirin in HF patients, which could help to prevent and predict complication development after LVAD implantation.

## 1. Introduction

The left ventricular assist device (LVAD) is one of the standard and alternative therapies for advanced heart failure (HF) patients when heart transplantation is not feasible. The device is implanted via bridge-to-transplantation (BTT) and destination therapy (DT), thereby prolonging and improving patients’ quality of life [1,2].

Despite the recent advances made in cardiology, patients still face adverse events, such as thrombosis, stroke, and bleeding [3]. These complications can reduce the life expectancy of patients with LVADs. For instance, pump thrombosis leads to pump exchange, after which a patient’s survival rate decreases [4,5]. Thrombosis complications can develop due to the impairment of the platelet function, which is caused by the high non-physiological shear stress (NPSS) at the blade region of the device’s rotary [6,7,8]. To prevent thrombosis development, a combination of antithrombotic drugs, such as warfarin (anticoagulant) and aspirin (antiplatelet), are prescribed for patients after device implantation [3,9,10]. However, patients can experience bleeding events due to the incorrect dosage of antithrombotic therapy [8]. On the other hand, NPSS and a low pulse pressure of continuous flow can lead to the development of bleeding events, followed by *platelet dysfunction* and the loss of the high-molecular-weight multimers of the von Willebrand factor (VWF). Subsequently, platelet dysfunction can be followed by the *loss of effective adhesion and aggregation* in HF patients with implanted devices [8,10,11].

Previous studies have shown that polymorphisms in the vitamin K epoxide reductase complex 1 (*VKORC1*) and cytochrome P450 2C9 (*CYP2C9*) genes could minimize complications due to anticoagulant (warfarin) therapy. The genetic variants of these genes influence the metabolism and action of warfarin and cause a 40–50% variability in dosage [2]. Thus, to some extent, it would be possible to eliminate the risks of the over- and under-coagulation of warfarin by correctly identifying personalized dosages of the drug based on one’s genetic polymorphisms [2,7,12].

To date, a proper dosage of aspirin based on genetic polymorphism has not been studied extensively in HF patients with implanted LVADs [2,12,13,14]. The salicylic acid form of aspirin after deacetylation is metabolized in two phases. In the first phase, the enzymes of cytochrome P450s play significant roles; in the second phase, the enzymes involved in glucuronidation [15] play significant roles. In the second phase of glucuronidation, *UGT1A6* is one of the main enzymes involved in aspirin metabolism [14]. As shown by Chen and colleagues (2007), the excretion of aspirin differed by genetic polymorphisms of *UGT1A6* [16]. The genetic polymorphism rs2070959 in the *UGT1A6* gene was associated with the varying levels of metabolic activity of the enzyme, leading to the faster or slower excretion of aspirin metabolites [7,13,14,16]. To our knowledge, only two studies—one by Topkara and colleagues (2016) [2] and another by Awad and colleagues (2015) [12]—have investigated the relations between aspirin metabolism and genetic polymorphisms in the *VKORC1* and *CYP2C9* genes in HF patients. However, the *UGT1A6* gene has not been studied in HF patients with implanted LVADs [14]. In a previous study, a polymorphism in rs2070959 in the *UGT1A6* gene showed no association in cardiovascular patients with gastrointestinal illness, even though this polymorphism was found to be associated with colon and colorectal cancer [14,17,18,19]

These studies suggest that genetic polymorphisms in these genes could provide insight into proper dosages for the antithrombotic therapy of warfarin/aspirin [20,21], which would, in turn, prevent complications in HF patients following LVAD implantation [2,14]. In the present study, we aim to characterize the influence of the genetic polymorphisms in the *UGT1A6* gene on complications in HF patients under antiplatelet therapy with implanted LVADs.

## 2. Materials and Methods

### 2.1. Participants

This retrospective study was performed in accordance with the Declaration of Helsinki. The Ethics Committee of National Laboratory Astana, Nazarbayev University (No. 16 from 11 March 2015) and the Ethics Committee of the National Research Cardiac Surgery Center (NRCC), Astana, Kazakhstan (No. 16 from 24 April 2015) approved the study [14]. All participants provided written informed consent before enrollment in the study. To protect the rights of patients and to protect their privacy, patients’ personal information was encoded and all the data were de-personalized during data collection. We recruited 100 HF patients implanted with LVADs as BTT and DT according to their medical indications from 2011 to 2016. These patients were the first participants of the LVAD programme at the NRCC in Kazakhstan since 2011. Two patients < 18 years old (9 and 16 years old) were excluded from the analysis. Patients were diagnosed with ischemic cardiomyopathy (ICM), dilated cardiomyopathy (DCM), hypertrophic cardiomyopathy (HCM), and valvular heart disease (VHD) (Table 1). Three types of LVADs were implanted such as HeartWare HVADs (*n* = 18, HW) (HeartWare Inc., Framingham MA, USA), HeartMate II (*n* = 34, HM2) (Thoratec Corporation, Pleasanton, CA, USA), and HeartMate III (*n* = 46, HM3) (St Jude Medical, Huntingdon, Cambridgeshire, UK). The follow-up period was three years from 2014 to 2017 with a median of 18 months. Venous blood samples collected in sterile vacutainers with K2EDTA were used to perform genetic analysis. The baseline demographic and clinical characteristics of HF patients are summarized in Table 1.

Following the standard clinical protocol guidelines from the Ministry of Healthcare of the Republic of Kazakhstan, patients were prescribed warfarin and aspirin antithrombotic therapy for the long term after device implantation. The dose of warfarin (2.99 ± 1.15 mg/day) was corrected to maintain the target international normalized ratio range (INR 2.25–3.25). The daily dose of aspirin (100 mg/day) was prescribed for HF patients according to their medical indication and ranged monthly in dynamics (before 14 days, as well as after 1, 3, 6, 12, and 18 months), unless there was a contraindication, such as ulcerative gastritis or liver cirrhosis. Furthermore, monthly aspirin therapy (100 mg/day) was coded as “1. with treatment” and “2. without treatment” for additional comparative analysis.

Post-implantation, 24 HF patients developed major adverse events such as thrombosis, bleeding, and infections. Three patients had both thrombosis and bleeding complications. Furthermore, patients were categorized into two groups according to the presence or absence of complication (thrombosis, bleeding, and infection) events for comparative analysis, as follows: Group 1—without complications (*n* = 74); Group 2—with complications (*n* = 24) (Table 1). Baseline demographic and clinical characteristics were compared between the two groups (Table 1). In addition, the effects of the monthly aspirin therapy (before 14 days, as well as after 1, 3, 6, 12, and 18 months) of HF patients were compared between the groups (Table 2).

### 2.2. Genetic Study

Genomic DNA was extracted from whole blood samples (200 µL) using the PureLinkTM Genomic DNA Mini Kit (Invitrogen, Carlsbad, CA, USA) following the manufacturer’s instructions. The concentration and purity of the DNA were measured on a Nanodrop 2000 (ThermoScientific, Waltham, MA, USA). For the genotyping of the genomic DNA, we performed a real-time polymerase chain reaction (qPCR) with allele discrimination using the TaqMan Real-Time PCR Assay on a 7900HT Fast Real-Time PCR System (Applied Biosystems, Waltham, MA, USA).

We genotyped 98 patients for 21 single-nucleotide polymorphisms (SNPs) in genes in the warfarin and aspirin interactive pathways, and tested them for association with dose requirement and with cardiovascular events [14]. We selected SNPs in or flanking the four candidate genes VKORC1, CYP2C9, GGCX, and CYP4F2 for their reported association with warfarin metabolism and previous studies in HF patients with implanted devices [2,12]. In addition, genetic polymorphisms in CYP2C19, ITGB3, ACSM2A, PTGS1, and UGT1A6 were examined as they interact with aspirin metabolism in cardiovascular diseases [15,16,17]. Moreover, polymorphisms of genes associated with thrombosis complications, such as MTHFR, FGB, F2, F7, F5, and F13A1 genes, were included as they are involved in the folate/homocysteine metabolism and coagulation system [14]. The list of SNPs and primers is summarized in Supplementary Table S1.

### 2.3. Statistical Analysis

Continuous variables are presented as mean *±* standard deviation (SD). The normality of continuous variables was tested using a Kolmogorov–Smirnov test. For continuous variables, the two groups were tested using Student’s *t*-test when normality was satisfied and the Mann–Whitney U test when normality was not satisfied. Categorical variables were presented as frequencies and percentages, and were compared using the Chi-square test or Fisher’s exact test. Sample size and power analysis were estimated using an online calculator (https://clincalc.com (accessed on 5 September 2021)) [14]. The sample size achieved 85% power with an alpha of 0.05. According to the sample size calculation results, each group needed to co-include 16 or more patients.

The Hardy–Weinberg equilibrium (HWE) was assessed using the chi-square test or Fisher’s exact test. A genetic association analysis between SNPs and HF patients with/without complications was performed and characterized according to odds ratios (ORs) with a 95% confidence interval (CI) and *p*-value [14]. Moreover, genetic association analysis was performed between polymorphism rs2070959 in the UGT1A6 gene and HF patients with/without aspirin treatment in monthly intake. Logistic regression analysis was performed using the web tool https://snpstats.net, accessed on 28 August 2024. The statistical analysis was processed using SPSS version 23 (SPSS, Chicago, IL, USA).

## 3. Results

### 3.1. Baseline Clinical Characteristics

Table 1 reports the baseline demographic and clinical characteristics of HF patients (*n* = 98) and the results of a comparative analysis between patients with and without complications. Most of the patients had diagnoses of ICM (*n* = 44) and DCM (*n* = 40) (Table 1). Device complications were developed after implantation, such as thrombosis in 13 cases (54.2%), bleeding in 14 cases (58.3%), and infection in 15 (62.5%) cases in the group of patients with complications (*p* = 0.0001, *p* = 0.0001, and *p* = 0.015, respectively). Post-implantation complications were more prevalent with implanted HM2 devices in 12 patients, and less occurred with the HW implanted device in *n* = 7 patients and with the HM3 implanted device in *n* = 5 patients. The mean duration of LVAD support was 29.6 ± 17.3 months in the study period (2011–2016) (Table 1).

Seventy-one (72.4%) patients reached the outcome measurement time, whereas twenty-seven (27.6%) patients did not reach the outcome measurement. Ten (10.2%) patients who reached the outcome measurement time had heart transplantation (BTT).

The initial dose of warfarin was 2.99 ± 1.15 mg/day, while the aspirin dose was 100 mg/day. Moreover, Table 2 summarizes a comparative analysis of monthly aspirin therapy frequency between patients with and without complications. On the 6th month, 49 patients (66.2%) were not receiving aspirin treatment in the group of patients without complications (Group 1), whereas 15 patients (62.5%) were receiving aspirin treatment in the group of patients with complications (Group 2) (*p* = 0.02).

### 3.2. Analysis of Genotyping

All HF patients (*n* = 98) in the group of patients with/without complications were genotyped for 21 selected SNPs (see Supplementary Table S1) [14]. The distributions of allelic and genotype frequencies of SNPs rs9934438 and rs9923231 in the *VKORC1* gene, rs5918 in the *ITGB3* gene, and rs2070959 in the *UGT1A6* gene showed significant differences between HF patient groups with and without complications (see Supplementary Table S2).

Moreover, polymorphisms rs9934438 and rs9923231 in the *VKORC1* gene, rs5918 in the *ITGB3* gene, and rs2070959 in the *UGT1A6* gene were significantly associated with complications of HF patients according to the logistic regression analysis (see Supplementary Table S3).

### 3.3. Genotype Association of rs2070959 in the UGT1A6 Gene with Aspirin Treatment

Logistic regression analysis was used to analyze the association between genetic polymorphisms of rs2070959 in the *UGT1A6* gene and HF patients with/without aspirin treatment in the monthly aspirin therapy (before 14 days, as well as after 1, 3, 6, 12, and 18 months) of all HF patients (*n* = 98) (Table 3). Polymorphism rs2070959 in the *UGT1A6* gene was considered in the analysis as it is involved in the metabolism of aspirin excretion.

Logistic regression analysis (adjusted for age, BMI, and sex) showed that polymorphism rs2070959 in the UGT1A6 gene is significantly associated with HF patients who were on aspirin treatment in the 12th month and without aspirin in the 18th month of therapy (Table 3). The GG genotype in the recessive and codominant model of rs2070959 in the UGT1A6 gene showed a significant association with aspirin treatment in the 12th month [OR (95% CI): 5.10 (1.31–19.87), *p* = 0.018 and (OR (95% CI): 4.50 (1.05–19.25), *p* = 0.054)]. On the other hand, the AG genotype in an over-dominant model of rs2070959 in the UGT1A6 gene showed a significant association without aspirin treatment in the 18th month [OR (95% CI): 0.33 (0.12–0.95), *p* = 0.032)].

### 3.4. Distribution of rs2070959 in the UGT1A6 Gene in the Group of Patients with Complications

The genotype distribution of rs2070959 in the UGT1A6 gene in monthly aspirin therapy was investigated in the HF patients with complications group for a more detailed analysis (*n* = 24) (Table 4). The distribution analysis of genotype polymorphism rs2070959 in the UGT1A6 gene showed statistically significant results after the 12th month of aspirin treatment. The frequency of the GG genotype was significantly higher in HF patients (*n* = 5) with aspirin treatment compared to patients without treatment. On the other hand, the frequency of the AA genotype was significantly higher in HF patients (*n* = 8) without aspirin treatment than in patients with aspirin treatment (57.1% versus 40.0%, *p* = 0.008).

## 4. Discussion

Our study aimed to investigate the influence of genetic polymorphism on the development of complications in HF patients with prescribed aspirin therapy (with/without treatment) with implanted LVADs. Previously, in our study, a comparative analysis was performed between groups of HF patients with/without complications after LVAD implantation. According to the research results, the genotypes of polymorphisms rs9934438 and rs9923231 in the *VKORC1* gene, rs5918 in the *ITGB3* gene, and rs2070959 in the *UGT1A6* gene were significantly associated with complications in HF patients (*p* < 0.05) [14]. Moreover, a comparative analysis was performed between the types of implanted LVADs (HW, HMII, and HM3) and the influence of the genetic polymorphisms on complication development, apart from the non-physiological shear stress of mechanical circulatory support devices. Our investigation revealed that polymorphisms of rs9923231 in the *VKORC1* and rs5918 in the *ITGB3* gene are significantly associated with HF patients (*p* < 0.05) [7]. The current study shows that the genotype polymorphism of rs2070959 in the *UGT1A6* gene was significantly associated with HF patients with/without aspirin treatment during the follow-up period (*p* < 0.05).

Aspirin and warfarin are the primary dual antithrombotic therapy that is prescribed to HF patients after device implantation for the prevention of device-related thromboembolic complications [3,10]. The influence of the genotype polymorphisms of the *VKORC1* and *CYP2C9* genes was very well studied in relation to warfarin dose variability in HF patients with LVADs [2,20]. Warfarin is a commonly prescribed anticoagulant that is difficult to use because of the wide inter-individual variation in dose requirements, the narrow therapeutic range, and the risk of serious bleeding. Device complications could be reduced and predicted by preventing the over- and under-coagulation of warfarin according to the genotyping test results for genetic polymorphisms of genes *VKORC1* and *CYP2C9* [20,21].

On the other hand, aspirin dose variability was not identified by genetic polymorphisms in HF patients despite developing complications with implanted LVADs such as non-surgical bleeding, gastrointestinal bleeding, and ischaemic and haemorrhagic stroke events [2,3,12]. Therefore, our research studied the genotype polymorphism rs2070959 of the UGT1A6 gene as it encodes the UDP-glucuronosyltransferase enzyme participating in aspirin metabolism. In our previous study, according to the regression analysis, the GG genotype in a recessive model of rs2070959 in the UGT1A6 gene showed a significant association with complications in HF patients (OR (95% CI): 4.40 (1.06–18.20), *p* = 0.044) (see Supplementary Table S3) [14]. Therefore, in the current study, polymorphism rs2070959 of the *UGT1A6* gene was studied more as it showed an association with complications of HF patients and had not been studied previously like the genotype polymorphisms of the *VKORC1* and *CYP2C9* genes [2,14]. Previously, van Oijen et al. investigated *UGT1A6* gene polymorphisms in cardiovascular patients with gastrointestinal complaints under aspirin treatment but did not show any significant associations [17].

The metabolic activities of the UDP-glucuronosyltransferase enzyme participating in the second phase of aspirin metabolism differentiate between genotype polymorphisms rs2070959 in the *UGT1A6* gene [16,22]. The salicylic acid form of aspirin excretes slowly with the wild-type AA genotype of rs2070959 in the *UGT1A6* gene, whereas with the presence of the mutant GG genotype, aspirin metabolites excrete faster due to the enzyme’s higher metabolic activity [13,14]. Hence, a higher aspirin dose should be prescribed for patients with the presence of the GG genotype due to the faster metabolic activity of the enzyme. On the contrary, patients with the AA genotype should be prescribed a lower aspirin dose due to the slower enzyme activity [14,16].

In the current study, according to the regression analysis (adjusted for age, BMI, and gender), we identified an association between the genotype polymorphism of rs2070959 in the *UGT1A6* gene and HF patients’ monthly aspirin therapy (with/without) to identify the influence of the genetic polymorphism (Table 3). According to the regression analysis, the mutant GG genotype of the polymorphism rs2070959 in the UGT1A6 gene was significantly associated with aspirin treatment in 26 HF patients in the 12th month of therapy [OR (95% CI): 5.10 (1.31–19.87), *p* = 0.018 and (OR (95% CI): 4.50 (1.05–19.25), *p* = 0.054)]. Therefore, the achieved results showed that the long-lasting treatment of aspirin for up to 12 months with the same dose (100 mg) was not effective for HF patients with the presence of the GG genotype of rs2070950 in the UGT1A6 gene due to the faster excretion of aspirin metabolites (Table 3).

Moreover, according to the distribution analysis, especially in the group of patients with complications (*n* = 24), the GG genotype of rs2070959 in the *UGT1A6* gene was significantly higher with aspirin treatment than without treatment (50.0% vs. 0%, *p* = 0.008) (Table 4). A higher frequency of the GG genotype with long-lasting aspirin therapy up to 12 months showed that 100 mg of aspirin was not an effective dose in the group of patients with complications. On the other hand, the wild-type AA genotype of rs2070959 in the *UGT1A6* gene was significantly higher in patients without aspirin treatment than in patients with aspirin treatment (57.1% vs. 40.0%, *p* = 0.008). Research results showed that aspirin treatment (100 mg) was high for eight patients (57.1%) with complications with the AA genotype, which led to the cessation of the treatment (*p* = 0.008). Incorrect doses of aspirin cause an increased/decreased inhibition of platelets via the acetylation isoform of cyclooxygenase-1 (COX-1) and cyclooxygenase -2 (COX-2), which leads to complication development [3,10,23].

However, no studies have been conducted on the effects of genetic polymorphisms on aspirin dosing; other clinical studies have been conducted on patients with LVADs aside from genetic research [24,25,26]. Previously, the European TRACE (STudy of Reduced AntiCoagulation/Antiplatelet Therapy in Patients with the HeartMatE II) multicenter study was performed on reduced antithrombotic therapy. The multicenter TRACE study identified that bleeding complications can be reduced by single anticoagulant therapy (INR 2.3) and with removed antiplatelet therapy in patients with HMII devices [27]. On the other hand, the US-TRACE study found that a higher risk of thrombosis complications could follow a decrease in the anti-thrombotic treatment in patients with implanted HMII devices. Additionally, bleeding complications may often occur in HF patients who are susceptible to such incidents [25]. Moreover, the placebo-controlled ARIES-HM3 (Antiplatelet Removal and Hemocompatibility Events With the HeartMate 3 Pump) trial demonstrated a reduction in bleeding and thrombosis complications in patients who did not receive aspirin therapy but were treated with warfarin anticoagulant [10]. On the contrary, the ADVANCE trial on the HeartWare HVAD prescribed 325 mg/day of aspirin to exclude pump thrombosis. However, the old generation of devices was associated with an increased risk of pump thrombosis and stroke events even with a lower aspirin dose (81 mg) [28,29]. According to the research conclusion of various clinical trials, it was shown that the impact of aspirin therapy can vary depending on the type of LVAD. For instance, discontinuing aspirin or reducing its dosage might increase the risk of thrombotic complications [3].

The shear stress of the device affects hemostatic function, which is followed by acquired von Willebrand factor (aVWF) syndrome, as well as the shedding and damage of the platelet glycoprotein receptors such as GPIb*α*, GPVI, and GPIIb/IIIa. Platelet dysfunction leads to the development of bleeding and thrombosis complications [6,7]. Genetically inherited platelet dysfunction is also one of the factors that might cause complications. For instance, in our previous study, we performed a comparative analysis between three type of device (HW, HMII, and HM3) and studied the influence of the genetic polymorphisms on complication development [7]. According to the research results, HF patients had more complications, increased changes in biochemical parameters (LDH, hemoglobin, hematocrit, etc.), and found that the TC genotype of polymorphism rs5918 in the ITGB3 gene was significantly higher with thrombosis complications in HF patients with implanted HMII devices than with HM3 devices (*p* < 0.05). Our study found that the identification of the TC genotype of rs5918 in the ITGB3 gene might prevent complication development at the pre-/post-LVAD implantation period as it encodes platelet receptor GPIIIa, which causes thrombosis/bleeding complications due to the inherited platelet receptor dysfunction [7]. Inherited platelet receptor dysfunction is one of the factors that might cause complication development in HF patients, alongside the antithrombotic therapy and shear stress of the device [7].

## 5. Conclusions

In summary, this study reports that genotyping for polymorphisms of *ITGB3*, *UGT1A6*, and *VKORC1* genes could help to prevent and predict complication (thrombosis/bleeding) development in HF patients with implanted LVADs, which will improve the quality of life, and reduce the morbidity and mortality rate. Our study found that personalized treatment for HF patients according to their genotypes in VKORC1 and UGT1A6 gene polymorphisms could help clinicians to identify recommended doses of antiplatelet (aspirin) and anticoagulant (warfarin) drugs.

Our studies conclude that LVAD complications develop not only due to the incorrect dose of antithrombotic drugs and the influence of the device’s non-physiological shear stress (NPSS), but also due to the inherited individual genetic characteristics of HF patients. Currently, next-generation sequencing technologies such as whole exome and whole genome sequencing would be helpful in preventing and predicting complications in HF patients if performed before LVAD implantation.

## 6. Limitations

The study was retrospective and included the first 98 HF patients with implanted LVADs who were genotyped for 21 selected SNPs. The small sample size limited us from performing more statistical analyses. As our study was retrospective, the aspirin dose could not be prescribed according to the genotyping test results for the genetic polymorphism of the UGT1A6 gene. In the future, more studies, including a higher number of patients and additional polymorphisms, will be needed to clarify the role of genetics in complication prediction and prevention in HF patients with implanted LVADs.

## Figures and Tables

**Table 1:** Baseline demographic and clinical characteristics of patients and comparison of patients with/without complications.

	Characteristic	Total HF Patients (*n* =98)	Comparison Between HF Patients		
			Group 1—Without Complications (*n* = 74)	Group 2—With Complications (*n* = 24)	*p* Value
1	Age, years	52.7 ± 11.0	52.5 ± 11.3	53.4 ± 10.1	0.92
	Sex, *n* (%)				
2	Male	92 (93.9)	71 (95.9)	21 (87.5)	0.16
	Female	6 (6.1)	3 (4.1)	3 (12.5)	
3	Race, *n* (%)				
	Asian	77 (78.6)	56 (75.7)	21 (87.5)	0.27
	Caucasian	21 (21.4)	18 (24.3)	3 (12.5)	
4	Body weight, kg	79.8 ± 13.9	80.0 ± 12.2	79.3 ± 18.5	0.86
5	Height, cm	169.8 ± 6.36	170.0 ± 6.08	168.9 ± 7.24	0.46
6	BMI, kg/m	27.7 ± 4.5	27.7 ± 4.10	27.6 ± 5.66	0.97
9	Smoking, *n* (%)				
	Smokers	58 (59.2)	46 (62.2)	12 (50.0)	0.34
	Non-smokers	40 (40.8)	28 (37.8)	12 (50.0)	
	Arterial blood pressure (mm Hg)				
7	Systolic	104.8 ± 15.5	105.0 ± 15.7	104.0 ± 15.0	0.99
8	Diastolic	71.2 ± 10.3	70.9 ± 10.7	72.1 ± 9.35	0.33
	Left ventricular ejection fraction, %	21.9 ± 5.36	21.9 ± 4.95	22.0 ± 6.56	0.97
14	Diagnosis, *n* (%)				
	ICM	44 (44.9)	36 (48.6)	8 (33.3)	0.10
	DCM	40 (40.8)	25 (33.8)	15 (62.5)	
	HCM	11 (11.2)	10 (13.5)	1 (4.2)	
	VHD	3 (3.1)	3 (4.1)	0	
	NYHA class, *n* (%)				
	I	1 (1.0)	1 (1.4)	0	0.58
	II	1 (1.0)	1 (1.4)	0	
	III	2 (2.0)	1 (1.4)	1 (4.2)	
	IV	26 (26.5)	17 (23.0)	9 (37.5)	
	IIIA	34 (34.7)	27 (36.5)	7 (29.2)	
	IIIB	34 (34.7)	27 (36.5)	7 (29.2)	
10	INR				
	Basic INR	1.21 ± 0.36	1.19 ± 0.37	1.26 ± 0.33	0.11
	Target INR	2.39 ± 0.26	2.36 ± 0.24	2.46 ± 0.32	0.06
11	Heart transplantation, *n* (%)				
	Received HT	10 (10.2)	6 (8.1)	4 (16.7)	0.25
	Did not receive HT	88 (89.8)	68 (91.9)	20 (83.3)	
12	Device type, *n* (%)				
	HW	18 (18.4)	11 (14.9)	7 (29.2)	0.01 *
	HM2	34 (34.7)	22 (29.7)	12 (50.0)	
	HM3	46 (46.9)	41 (55.4)	5 (20.8)	
13	Warfarin dose, mg/day (1st month)	2.99 ± 1.15	3.01 ± 1.04	2.92 ± 1.46	0.29
	Aspirin, 100 mg/day, Yes/No (1st month), *n* (%)	79 (80.6)/19 (19.4)	62 (83.8)/12 (16.2)	17 (70.8)/7 (29.2)	0.23
15	Duration of LVAD support until outcome, from 2011 to 2016, *n* = 36 (in months)	29.6 ± 17.3	29.1 ± 17.6 (*n* = 21)	30.3 ± 17.5 (*n* = 15)	0.84
16	Patients’ achieved outcome until 2017, *n* (%)				
	Survived	71 (72.4)	58 (78.4)	13 (54.2)	0.03 *
	Did not survive	27 (27.6)	16 (21.6)	11 (45.8)	
17	Thrombosis complications, *n* (%)				
	Yes	13 (13.3)	0	13 (54.2)	0.0001 *
	No	85 (86.7)	74 (100)	11 (45.8)	
18	Bleeding complications, *n* (%)				
	Yes	14 (14.3)	0	14 (58.3)	0.0001 *
	No	84 (85.7)	74 (100)	10 (41.7)	
19	Infections, *n* (%)				
	Yes	39 (39.8)	24 (32.4)	15 (62.5)	0.015 *
	No	59 (60.2)	50 (67.6)	9 (37.5)	
20	Stroke, *n* (%)				
	No Stroke	78 (79.6)	60 (81.1)	18 (75.0)	0.57
	Hemorrhagic stroke	8 (8.2)	5 (6.8)	3 (12.5)	
	Ischemic stroke	12 (12.2)	9 (12.2)	3 (12.5)	
21	Myocardial infarction, *n* (%)				
	Yes	44 (44.9)	36 (48.6)	8 (33.3)	0.24
	No	54 (55.1)	38 (51.4)	16 (66.7)	

Continuous variables are presented as mean ± SD and categorical variables as *n* (%). HF patients—heart failure patients. The significant *p*-value (*p* < 0.05) is labelled with an asterisk (*). BMI—body mass index; ICM—ischemic cardiomyopathy; DCM—dilated cardiomyopathy; HCM—hypertrophic cardiomyopathy; VHD—valvular heart disease; HT—heart transplantation; NYHA—New York Heart Association; INR—international normalized ratio; HW—HeartWare HVAD; HM2—HeartMate II; HM3—HeartMate III.

**Table 2:** Aspirin intake in groups of patients with/without complications.

	Monthly Therapy of Aspirin, 100 mg/day	Total HF Patients (*n* = 98)	Comparison Between HF Patients		
			Group 1—Without Complications (*n* = 74)	Group 2—With Complications (*n* = 24)	*p*-Value
1	Before 14 days of implantation				
	With treatment	9 (9.2)	7(9.5)	2 (8.3)	1
	Without treatment	89(90.8)	67 (90.5)	22 (91.7)	
2	In the 1st month				
	With treatment	79 (80.6)	62 (83.8)	17 (70.8)	0.27
	Without treatment	19 (19.4)	12 (16.2)	7 (29.2)	
3	3rd month				
	With treatment	38 (38.8)	30 (40.5)	8 (33.3)	0.7
	Without treatment	60 (61.2)	44 (59.5)	16 (66.7)	
4	6th month				
	With treatment	40 (40.8)	25 (33.8)	15 (62.5)	0.02 *
	Without treatment	58 (59.2)	49 (66.2)	9 (37.5)	
5	12th month				
	With treatment	26 (26.5)	16 (21.6)	10 (41.7)	0.09
	Without treatment	72 (73.5)	58 (78.4)	14 (58.3)	
6	18th month				
	With treatment	21 (21.4)	13 (17.6)	8 (33.3)	0.18
	Without treatment	77 (78.6)	61 (82.4)	16 (66.7)	

HF patients—heart failure patients. The significant *p*-value (*p* < 0.05) is labelled with an asterisk (*).

**Table 3:** Association analysis of genotype polymorphism rs2070959 in the *UGT1A6* gene with/without aspirin therapy in HF patients (adjusted for age, BMI, and gender).

Monthly Therapy of Aspirin	Model	Genotype	Aspirin Therapy, 100 mg/day		OR (95% CI)	*p*-Value	AIC	BIC
			Without Treatment, *n* = 89	With Treatment, *n* = 9				
Before 14 days of implantation	Codominant	A/A	38 (42.7%)	2 (22.2%)	1.00	0.46	64.6	72.3
		A/G	42 (47.2%)	6 (66.7%)	2.71 (0.52–14.27)			
		G/G	9 (10.1%)	1 (11.1%)	2.11 (0.17–25.93)			
	Dominant	A/A	38 (42.7%)	2 (22.2%)	1.00	0.22	62.6	67.8
		A/G-G/G	51 (57.3%)	7 (77.8%)	2.61 (0.51–13.26)			
	Recessive	A/A-A/G	80 (89.9%)	8 (88.9%)	1.00	0.93	64.1	69.3
		G/G	9 (10.1%)	1 (11.1%)	1.11 (0.12–9.93)			
	Over-dominant	A/A-G/G	47 (52.8%)	3 (33.3%)	1.00	0.26	62.9	68
		A/G	42 (47.2%)	6 (66.7%)	2.24 (0.53–9.51)			
	Log-additive	---	---	---	1.65 (0.58–4.64)	0.35	63.2	68.4
			Without treatment, *n* = 19	With treatment, *n* = 79				
After 1st month	Codominant	A/A	9 (47.4%)	31 (39.2%)	1.00	0.13	98.3	106
		A/G	6 (31.6%)	42 (53.2%)	2.03 (0.65–6.31)			
		G/G	4 (21.1%)	6 (7.6%)	0.44 (0.10–1.89)			
	Dominant	A/A	9 (47.4%)	31 (39.2%)	1.00	0.52	100	105.1
		A/G-G/G	10 (52.6%)	48 (60.8%)	1.39 (0.51–3.82)			
	Recessive	A/A-A/G	15 (79%)	73 (92.4%)	1.00	0.11	97.8	103
		G/G	4 (21.1%)	6 (7.6%)	0.31 (0.08–1.23)			
	Over-dominant	A/A-G/G	13 (68.4%)	37 (46.8%)	1.00	0.088	97.5	102.6
		A/G	6 (31.6%)	42 (53.2%)	2.46 (0.85–7.12)			
	Log-additive	---	---	---	0.88 (0.41–1.90)	0.75	100.3	105.5
			Without treatment, *n* = 60	With treatment, *n* = 38				
After 3rd month	Codominant	A/A	23 (38.3%)	17 (44.7%)	1.00	0.39	135	142.8
		A/G	29 (48.3%)	19 (50%)	0.89 (0.38–2.08)			
		G/G	8 (13.3%)	2 (5.3%)	0.34 (0.06–1.80)			
	Dominant	A/A	23 (38.3%)	17 (44.7%)	1.00	0.53	134.5	139.7
		A/G-G/G	37 (61.7%)	21 (55.3%)	0.77 (0.34–1.75)			
	Recessive	A/A-A/G	52 (86.7%)	36 (94.7%)	1.00	0.18	133.1	138.2
		G/G	8 (13.3%)	2 (5.3%)	0.36 (0.07–1.80)			
	Over-dominant	A/A-G/G	31 (51.7%)	19 (50%)	1.00	0.87	134.8	140
		A/G	29 (48.3%)	19 (50%)	1.07 (0.47–2.41)			
	Log-additive	---	---	---	0.70 (0.37–1.34)	0.28	133.7	138.9
			Without treatment, *n* = 58	With treatment, *n* = 40				
After 6th month	Codominant	A/A	22 (37.9%)	18 (45%)	1.00	0.23	135.6	143.4
		A/G	32 (55.2%)	16 (40%)	0.61 (0.26–1.45)			
		G/G	4 (6.9%)	6 (15%)	1.83 (0.45–7.51)			
	Dominant	A/A	22 (37.9%)	18 (45%)	1.00	0.48	136	141.2
		A/G-G/G	36 (62.1%)	22 (55%)	0.75 (0.33–1.69)			
	Recessive	A/A-A/G	54 (93.1%)	34 (85%)	1.00	0.2	134.9	140
		G/G	4 (6.9%)	6 (15%)	2.38 (0.63–9.06)			
	Over-dominant	A/A-G/G	26 (44.8%)	24 (60%)	1.00	0.14	134.3	139.5
		A/G	32 (55.2%)	16 (40%)	0.54 (0.24–1.23)			
	Log-additive	---	---	---	1.03 (0.55–1.91)	0.94	136.5	141.7
			Without treatment, *n* = 72	With treatment, *n* = 26				
After 12th month	Codominant	A/A	30 (41.7%)	10 (38.5%)	1.00	0.054 *	113.6	121.3
		A/G	38 (52.8%)	10 (38.5%)	0.79 (0.29–2.14)			
		G/G	4 (5.6%)	6 (23.1%)	4.50 (1.05–19.25)			
	Dominant	A/A	30 (41.7%)	10 (38.5%)	1.00	0.78	117.3	122.5
		A/G-G/G	42 (58.3%)	16 (61.5%)	1.14 (0.46–2.86)			
	Recessive	A/A-A/G	68 (94.4%)	20 (76.9%)	1.00	0.018 *	111.8	117
		G/G	4 (5.6%)	6 (23.1%)	5.10 (1.31–19.87)			
	Over-dominant	A/A-G/G	34 (47.2%)	16 (61.5%)	1.00	0.21	115.8	121
		A/G	38 (52.8%)	10 (38.5%)	0.56 (0.22–1.40)			
	Log-additive	---	---	---	1.64 (0.82–3.28)	0.16	115.4	120.6
			Without treatment, *n* = 77	With treatment, *n* = 21				
After 18th month	Codominant	A/A	29 (37.7%)	11 (52.4%)	1.00	0.076	102.7	110.4
		A/G	42 (54.5%)	6 (28.6%)	0.38 (0.13–1.13)			
		G/G	6 (7.8%)	4 (19.1%)	1.76 (0.42–7.44)			
	Dominant	A/A	29 (37.7%)	11 (52.4%)	1.00	0.23	104.4	109.5
		A/G-G/G	48 (62.3%)	10 (47.6%)	0.55 (0.21–1.45)			
	Recessive	A/A-A/G	71 (92.2%)	17 (81%)	1.00	0.16	103.8	109
		G/G	6 (7.8%)	4 (19.1%)	2.78 (0.71–10.97)			
	Over-dominant	A/A-G/G	35 (45.5%)	15 (71.4%)	1.00	0.032 *	101.3	106.4
		A/G	42 (54.5%)	6 (28.6%)	0.33 (0.12–0.95)			
	Log-additive	---	---	---	0.92 (0.43–1.95)	0.83	105.8	111

The *p*-value (*p* < 0.05) numbers with statistical significance are labelled with an asterisk (*). AIC—Akaike information criterion; BIC—Bayesian information criterion.

**Table 4:** Distribution of genotype polymorphism rs2070959 in the *UGT1A6* gene in HF patients (with complications) with prescribed aspirin therapy.

№	Aspirin Therapy, 100 mg/day (Monthly)	Genotypes of Polymorphism rs2070959, *n* = 24			
		AA, *n* = 12	AG, *n* = 7	GG, *n* = 5	*p*-Value
1	Before 14 days of implantation, *n* (%)				
	With treatment	1 (50.0)	1 (50.0)	0	1
	Without treatment	11 (50.0)	6 (27.3)	5 (22.7)	
2	After 1st month, *n* (%)				
	With treatment	9 (52.9)	6 (35.3)	2 (11.8)	0.26
	Without treatment	3 (42.9)	1 (14.3)	3 (42.9)	
3	After 3rd month, *n* (%)				
	With treatment	5 (62.5)	2 (25.0)	1 (12.5)	0.741
	Without treatment	7 (43.8)	5 (31.3)	4 (25.0)	
4	After 6th month, *n* (%)				
	With treatment	7 (46.7)	4 (26.7)	4 (26.7)	0.74
	Without treatment	5 (55.6)	3 (33.3)	1 (11.1)	
5	After 12th month, *n* (%)				
	With treatment	4 (40.0)	1 (10.0)	5 (50.0)	0.008 *
	Without treatment	8 (57.1)	6 (42.9)	0 (0)	
6	After 18th month, *n* (%)				
	With treatment	6 (75.0)	0 (0)	2 (25.0)	0.09
	Without treatment	6 (37.5)	7 (43.8)	3 (18.8)	

The *p*-value (*p* < 0.05) numbers with statistical significance are labelled with an asterisk (*).

## Data Availability

Please contact National Laboratory Astana (via phone or mail) to access confidential data. The data underlying the results presented in the study are available from the authors—phone number: +77172706501; mail: akilzhanova@nu.edu.kz.

## Supplementary Materials

The following supporting information can be downloaded at: https://www.mdpi.com/article/10.3390/jcm14010035/s1, Table S1: List of SNP characteristics. Table S2. The distributions of allelic and genotype frequencies of 21 SNPs between HF patients with/without complications. Table S3. Association analysis of SNPs for LVAD complications in HF patient groups with/without complications.
