# Supplementary material for: The Impact of Genetic Polymorphism on Complication Development in Heart Failure Patients
Source: J Clin Med. 2024 Dec 25;14(1):35. doi: 10.3390/jcm14010035 (PMC11721630; doi:10.3390/jcm14010035)
Supplement: Supplementary file 1 [file jcm-14-00035-s001.zip › jcm-3286088-supplementary.pdf]

Supplementary Material

Table S1. List of SNP characteristics

	Gene	SNP rs number	Gene name	Context Sequence [VIC/FAM]
1	*VKORC1*1*	rs8050894	vitamin K epoxide reductase complex subunit 1	CCACATCCCCACCCGCAGGACGCTC[G/C]GTGATGAGCAGCTAGCTGGCTGTCA
2	*VKORC1*2*	rs9934438		CCCCGACCTCCCATCCTAGTCCAAG[A/G]GTCGATGATCTCCTGGCACCGGGCA
3	*VKORC1*3*	rs9923231		GATTATAGGCGTGAGCCACCGCACC[C/T]GGCCAATGGTTGTTTTTCAGGTCTT
4	*CYP2C9*2*	rs1799853	cytochrome P450 family 2 subfamily C member 9	GATGGGGAAGAGGAGCATTGAGGAC[C/T]GTGTTCAAGAGGAAGCCCGCTGCCT
5	*CYP2C9*3*	rs1057910		TGTGGTGCACGAGGTCCAGAGATAC[C/A]TTGACCTTCTCCCCACCAGCCTGCC
6	*CYP2C9*5*	rs28371686		TGCACGAGGTCCAGAGATACATTGA[C/G]CTTCTCCCCACCAGCCTGCCCCATG
7	*CYP2C19*2*	rs4244285	cytochrome P450 family 2 subfamily C member 19	TTCCCACTATCATTGATTATTTCCC[A/G]GGAACCCATAACAAATTACTTAAAA
8	*CYP2C19*3*	rs4986893		ACATCAGGATTGTAAGCACCCCCTG[A/G]ATCCAGGTAAGGCCAAGTTTTTTGC
9	*ITGB3*	rs5918	integrin subunit beta 3	GCTCCTGTCTTACAGGCCCTGCCTC[C/T]GGGCTCACCTCGCTGTGACCTGAAG
10	*GGCX*	rs11676382	gamma-glutamyl carboxylase	CTCTCCCCAGGGGAAAGTTACCAAG[C/G]TTGCCAACATATGATGGCAATGACA
11	*CYP4F2*	rs2108622	cytochrome P450 family 4 subfamily F member 2	CCCCGCACCTCAGGGTCCGGCCACA[C/T]AGCTGGGTTGTGATGGGTTCCGAAA
12	*UGT1A6*	rs2070959	UDP glucuronosyltransferase family 1 member A9	GGGTTTTCCGTGTTCCCTGGAGCAT[A/G]CATTCAGCAGAAGCCCAGACCCTGT
13	*ACSM2A*	rs1133607	acyl-CoA synthetase medium-chain family member 2A	GTGAAGGCATTTGTGGTCCTGGCCT[T/C]GCAGTTCCTGTCCCATGACCCAGAA
14	*PTGS1*	rs3842787	prostaglandin-endoperoxide synthase 1	TTCTTGCTGTTCCTGCTCCTGCTCC[C/T]GCCGCTCCCCGTCCTGCTCGCGGAC
15	*F5*	rs6025	coagulation factor V	TCAAGGACAAAATACCTGTATTCCT[C/T]GCCTGTCCAGGGATCTGCTCTTACA
16	*F13A1*	rs5985	coagulation factor XIII A chain	TGCAGGTTGACGCCCCGGGGCACCA[A/C]GCCCTGAAGCTCCACTGTGGGCAGG
17	*F2*	rs1799963	coagulation factor II, thrombin	GTTCCCAATAAAAGTGACTCTCAGC[A/G]AGCCTCAATGCTCCCAGTGCTATTC
18	*F7*	rs6046	coagulation factor VII	GGAGGCCCACATGCCACCCACTACC[A/G]GGGCACGTGGTACCTGACGGGCATC

19	*FGB*	rs1800790	fibrinogen beta chain	ATATAACATTACTATTGATTTTAAT[A/G]GCCCCTTTTGAAATAGAATTATGTC
20	*MTHFR*1*	rs1801133	methylenetetrahydrofolate reductase (NAD(P)H)	GAAAAGCTGCGTGATGATGAAATCG[G/A]CTCCCGCAGACACCTTCTCCTTCAA
21	*MTHFR*2*	rs1801131		AAGAACGAAGACTTCAAAGACACTT[G/T]CTTCACTGGTCAGCTCCTCCCCCCA

Table S2. The distributions of allelic and genotype frequencies of 21 SNPs between HF patients with/without complications

	Gene	SNP rs number	Genotype	Group 1 - without complications, No. (%)	Allele frequency in Group 1	Group 2 -with complications, No. (%)	Allele frequency in Group 2	*P* value
1	VKORC1*1	rs8050894	CC	41 (55.4)	C : G = 0.68 : 0.32	14 (58.3)	C : G = 0.71 : 0.29	1
			CG	18 (24.3)		6 (25.0)		
			GG	15 (20.3)		4 (16.7)		
			C	100		34		
			G	48		14		
2	VKORC1*2	rs9934438	GG	14 (18.9)	G : A = 0.39 : 0.61	0	G : A = 0.35 : 0.65	0.008*
			GA	29 (39.2)		17 (70.8)		
			AA	31 (41.9)		7 (29.2)		
			G	57		17		
			A	91		31		
3	VKORC1*3	rs9923231	CC	14 (18.9)	C : T = 0.40 : 0.60	0	C : T = 0.35 : 0.65	0.012*
			CT	31 (41.9)		17 (70.8)		
			TT	29 (39.2)		7 (29.2)		
			C	59		17		
			T	89		31		
4	CYP2C9*2	rs1799853	CC	70 (94.6)	C : T = 0.97 : 0.03	21 (87.5)	C : T = 0.94 : 0.06	0.357
			CT	4 (5.4)		3 (12.5)		
			TT	0		0		
			C	144		45		
			T	4		3		
5	CYP2C9*3	rs1057910	AA	68 (91.9)	A : C = 0.96 : 0.04	23 (95.8)	A : C = 0.98 : 0.02	1
			AC	6 (8.1)		1 (4.2)		
			CC	0		0		
			A	142		47		
			C	6		1		
6	CYP2C9*5	rs28371686	CC	74 (100)	C : G = 1.000 : 0.000	24 (100)	C : G = 1.000 : 0.000	N/A
			CG	0		0		
			GG	0		0		
			C	148		48		
			G	0		0		
7	CYP2C19*2	rs4244285	GG	52 (70.3)	G : A = 0.84 : 0.16	19 (79.2)	G : A = 0.90 : 0.10	0.772
			GA	20 (27.0)		5 (20.8)		
			AA	2 (2.7)		0		
			G	124		43		

			A	24		5		
8	CYP2C19*3	rs4986893	GG	70 (94.6)	G : A = 0.97 : 0.03	23 (95.8)	G : A = 0.98 : 0.02	1
			GA	4 (5.4)		1 (4.2)		
			AA	0		0		
			G	144		47		
			A	4		1		
9	ITGB3	rs5918	TT	42 (56.8)	T : C = 0.70 : 0.30	6 (25.0)	T : C = 0.56 : 0.44	0.005*
			TC	19 (25.7)		15 (62.5)		
			CC	13 (17.6)		3 (12.5)		
			T	103		27		
			C	45		21		
10	GGCX	rs11676382	CC	70 (94.6)	C : G = 0.97 : 0.03	24 (100)	C : G = 1.000 : 0.000	0.677
			CG	3 (4.0)		0		
			GG	1 (1.4)		0		
			C	143		48		
			G	5		0		
11	CYP4F2	rs2108622	CC	47 (63.5)	C : T = 0.78 : 0.22	13 (54.2)	C : T = 0.71 : 0.29	0.501
			CT	22 (29.7)		8 (33.3)		
			TT	5 (6.8)		3 (12.5)		
			C	116		34		
			T	32		14		
12	UGT1A6	rs2070959	AA	28 (37.8)	A : G = 0.66 : 0.34	12 (50.0)	A : G = 0.65: 0.35	0.03*
			AG	41 (55.4)		7 (29.2)		
			GG	5 (6.8)		5 (20.8)		
			A	97		31		
			G	51		17		
13	ACSM2A	rs1133607	CC	44 (59.5)	C : T = 0.78 : 0.22	15 (62.5)	C : T = 0.79 : 0.21	1
			CT	27 (36.5)		8 (33.3)		
			TT	3 (4.0)		1 (4.2)		
			C	115		38		
			T	33		10		
14	PTGS1	rs3842787	CC	72 (97.3)	C : T = 0.99 : 0.01	23 (95.8)	C : T = 0.98 : 0.02	1
			CT	2 (2.7)		1 (4.2)		
			TT	0		0		
			C	146		47		
			T	2		1		
15	F5	rs6025	CC	73 (98.6)	C : T = 0.99 : 0.01	24 (100)	C : T = 1.000 : 0.000	1
			CT	1 (1.4)		0		
			TT	0		0		
			C	147		48		
			T	1		0		
16	F13A1	rs5985	CC	53 (71.6)	C : A = 0.84 : 0.16	19 (79.2)	C : A = 0.90 : 0.10	0.879
			CA	19 (25.7)		5 (20.8)		
			AA	2 (2.7)		0%		
			C	125		43		
			A	23		5		
17	F2	rs1799963	GG	74 (100)	G : A = 1.000 : 0.000	24 (100)	G ; A = 1.000 : 0.000	N/A
			GA	0		0		
			AA	0		0		
			G	148		48		
			A	0		0		
18	F7	rs6046	GG	57 (77.0)		18 (75.0)		1

			GA	15 (20.3)	G : A = 0.87 : 0.13	5 (20.8)	G : A = 0.85 : 0.15	
			AA	2 (2.7)		1 (4.2)		
			G	129		41		
			A	19		7		
19	FGB	rs1800790	GG	49 (66.2)	G : A = 0.82 : 0.18	16 (66.7)	G : A = 0.81 : 0.19	1
			GA	23 (31.1)		7 (29.2)		
			AA	2 (2.7)		1 (4.2)		
			G	121		39		
			A	27		9		
20	MTHFR*1	rs1801133	GG	37 (50.0)	G : A = 0.70 : 0.30	10 (41.7)	G : A = 0.65 : 0.35	0.742
			GA	30 (40.5)		11 (45.8)		
			AA	7 (9.5)		3 (12.5)		
			G	104		31		
			A	44		17		
21	MTHFR*2	rs1801131	TT	43 (58.1)	T : G = 0.76 : 0.24	11 (45.8)	T : G = 0.69 : 0.31	0.589
			TG	27 (36.5)		11 (45.8)		
			GG	4 (5.4)		2 (8.3)		
			T	113		33		
			G	35		15		

Group 1, without complications; Group 2, with complications; p-value of Hardy-Weinberg equilibrium test; the p-value (p<0.05) numbers with statistical significance are labelled with an asterisk (*) in bold

Table S3. Association analysis of SNPs for LVAD complications in HF patient groups with/without complications

**Association of VKORC1 rs9934438 genotypes with complication development in groups (adjusted for age, BMI and gender)**							
**Model**	**Genotype**	**Group 1 - without complications, No. (%)**	**Group 2 - with complications, No. (%)**	**OR (95% CI)**	**P-value**	**AIC**	**BIC**
Codominant	A/A	31 (41.9)	7 (29.2)	1.00	0.0015*	106.1	121.6
	G/A	29 (39.2)	17 (70.8)	2.69 (0.95-7.63)			
	G/G	14 (18.9)	0 (0)	0.00 (0.00-NA)			
Dominant	A/A	31 (41.9)	7 (29.2)	1.00	0.27	115.9	128.8
	G/A-G/G	43 (58.1)	17 (70.8)	1.73 (0.64-4.74)			
Recessive	A/A-G/A	60 (81.1)	24 (100)	1.00	0.0023	107.8	120.7
	G/G	14 (18.9)	0 (0)	0.00 (0.00-NA)			
Overdominant	A/A-G/G	45 (60.8)	7 (29.2)	1.00	0.0057*	109.4	122.4
	G/A	29 (39.2)	17 (70.8)	3.96 (1.42-11.02)			
Log-additive	---	---	---	0.85 (0.43-1.70)	0.65	116.9	129.8

**Association of VKORC1 rs9923231 genotypes with complication development in groups (adjusted for age, BMI and gender)**

Model	Genotype	Group 1 - without complications, No. (%)	Group 2 - with complications, No. (%)	OR (95% CI)	P-value	AIC	BIC
Codominant	T/T	29 (39.2)	7 (29.2)	1.00	0.0024	107	122.5
	C/T	31 (41.9)	17 (70.8)	2.36 (0.83-6.70)			
	C/C	14 (18.9)	0 (0)	0.00 (0.00-NA)			
Dominant	T/T	29 (39.2)	7 (29.2)	1.00	0.38	116.3	129.2
	C/T-C/C	45 (60.8)	17 (70.8)	1.56 (0.57-4.27)			
Recessive	T/T-C/T	60 (81.1)	24 (100)	1.00	0.0023	107.8	120.7
	C/C	14 (18.9)	0 (0)	0.00 (0.00-NA)			
Overdominant	T/T-C/C	43 (58.1)	7 (29.2)	1.00	0.011*	110.6	123.5
	C/T	31 (41.9)	17 (70.8)	3.55 (1.28-9.86)			
Log-additive	---	---	---	0.80 (0.40-1.62)	0.54	116.7	129.6

Association of ITGB3 rs5918 genotypes with complication development in groups (adjusted for age, BMI and gender)							
Model	Genotype	Group 1 - without complications, No. (%)	Group 2 - with complications, No. (%)	OR (95% CI)	P-value	AIC	BIC
Codominant	T/T	42 (56.8)	6 (25.0)	1.00	0.0056*	108.7	124.2
	T/C	19 (25.7)	15 (62.5)	5.37 (1.79-16.16)			
	C/C	13 (17.6)	3 (12.5)	1.47 (0.31-7.05)			
Dominant	T/T	42 (56.8)	6 (25.0)	1.00	0.0079*	110	123
	T/C-C/C	32 (43.2)	18 (75.0)	3.83 (1.35-10.89)			
Recessive	T/T-T/C	61 (82.4)	21 (87.5)	1.00	0.49	116.6	129.5
	C/C	13 (17.6)	3 (12.5)	0.62 (0.15-2.53)			
Overdominant	T/T-C/C	55 (74.3)	9 (37.5)	1.00	0.0014*	106.9	119.9
	T/C	19 (25.7)	15 (62.5)	4.83 (1.79-13.06)			
Log-additive	---	---	---	1.59 (0.84-2.99)	0.15	115	128

Association of UGT1A6 rs2070959 genotypes with complication development in groups (adjusted for age, BMI and gender)							
Model	Genotype	Group 1 - without complications, No. (%)	Group 2 - with complications, No. (%)	OR (95% CI)	P-value	AIC	BIC
Codominant	A/A	28 (37.8)	12 (50)	1.00	0.03	112.1	127.6
	A/G	41 (55.4)	7 (29.2)	0.40 (0.13-1.17)			
	G/G	5 (6.8)	5 (20.8)	2.67 (0.59-12.07)			
Dominant	A/A	28 (37.8)	12 (50)	1.00	0.29	116	128.9
	A/G-G/G	46 (62.2)	12 (50)	0.60 (0.23-1.56)			
Recessive	A/A-A/G	69 (93.2)	19 (79.2)	1.00	0.044*	113	125.9
	G/G	5 (6.8)	5 (20.8)	4.40 (1.06-18.20)			
Overdominant	A/A-G/G	33 (44.6)	17 (70.8)	1.00	0.02*	111.7	124.6
	A/G	41 (55.4)	7 (29.2)	0.32 (0.11-0.87)			
Log-additive	---	---	---	1.08 (0.51-2.29)	0.84	117	130

The p-value ($p < 0.05$) numbers with statistical significance are labelled with an asterisk (*); AIC, Akaike Information Criterion; BIC, Bayesian Information Criterion
